# Hipertensão Arterial em Trabalhadores: O Efeito Cumulativo das Dimensões da Atividade Física sobre esse Agravo

**DOI:** 10.36660/abc.20190065

**Published:** 2020-05-22

**Authors:** Uelito Everaldo Souza Ribeiro, Rita de Cassia Pereira Fernandes

**Affiliations:** 1 Universidade Federal da Bahia Programa de Pós-Graduação em Saúde, Ambiente e Trabalho SalvadorBA Brasil Universidade Federal da Bahia - Programa de Pós-Graduação em Saúde, Ambiente e Trabalho, Salvador, BA - Brasil; 2 Universidade Federal da Bahia Faculdade de Medicina da Bahia SalvadorBA Brasil Universidade Federal da Bahia - Faculdade de Medicina da Bahia, Salvador, BA – Brasil

**Keywords:** Hipertensão, Trabalhadores, Serviço de Limpeza Urbana, Atividade Motora, Antropometria, Esforço Físico, Trabalho, Fatores Socioeconômicos, Estilo de Vida

## Abstract

**Fundamento:**

A atividade física, em diferentes dimensões (ocupacional, doméstica e de lazer), pode ter papel relevante sobre a hipertensão arterial (HA). Praticar atividade física apenas no lazer, ou isoladamente nas outras dimensões, pode ser insuficiente para o controle efetivo da HA.

**Objetivo:**

Analisar o efeito isolado e cumulativo das dimensões da atividade física sobre a ocorrência de hipertensão arterial em trabalhadores.

**Métodos:**

Estudo transversal com 1.070 trabalhadores de Limpeza Urbana e Indústria de Calçados, na Bahia, utilizando questionário aplicado por entrevistador acerca de aspectos sociodemográficos, ocupacionais, de estilo de vida e morbidade hipertensiva.

**Foram medidos:**

peso, altura, circunferência abdominal e pressão arterial. Caso de HA: pressão arterial sistólica ≥140 ou diastólica ≥90, ou tratamento regular de Hipertensão. Foram investigadas as dimensões ocupacional, doméstica e de lazer da atividade física. Realizou-se análise multivariada com Regressão de Cox para estudos transversais.

**Resultados:**

A prevalência de HA foi de 24%, sendo 37% dentre pacientes com idades entre 35 e 44 anos, e 51% entre 45 e 54 anos. O modelo multivariado evidenciou que ativos em uma dimensão ou em nenhuma tiveram 62% mais HA, e um valor 25% mais elevado de HA foi observado dentre aqueles trabalhadores ativos em duas das três dimensões, na comparação com os ativos nas três dimensões. Sexo masculino, maior idade (> 31 anos) e excesso de peso se associaram à HA, com razões de prevalência de 1,62, 2,10 e 2,26, respectivamente.

**Conclusões:**

Verificou-se efeito cumulativo das dimensões da atividade física sobre a ocorrência de HA. Classificar sujeitos ativos no trabalho ou no ambiente doméstico como inativos ao se basear apenas na dimensão do lazer pode implicar em erro metodológico. (Arq Bras Cardiol. 2020; 114(5):755-761)

## Introdução

Hipertensão arterial (HA) é uma doença crônica definida por níveis pressóricos maiores ou iguais a 140/90 mmHg, representando um dos fatores de risco para doenças cardiovasculares. Por outro lado, sabe-se que a atividade física (AF) ausente ou insuficiente é risco modificável nesse agravo.^1,2^

Todo movimento produzido pela musculatura esquelética que gere energia maior que o estado de repouso é AF. Considera-se ativo o indivíduo que pratica no mínimo 30 minutos de AF com intensidade moderada, cinco dias por semana. Isso não inclui apenas o lazer, ao contrário, a literatura ressalta a importância de se abordar a AF em diferentes dimensões: lazer (AFL), ocupacional (AFO), doméstica (AFD) e de deslocamento.^3,4^

O Brasil, signatário do Plano de Prevenção das Doenças Crônicas não Transmissíveis da Organização Mundial da Saúde, comprometeu-se com a redução relativa de 25% da prevalência de HA e 10% de AF insuficiente, até o ano de 2025. Esse desafio explicita a relevância da HA como problema de saúde.^5,6^

Embora a associação negativa entre AF e Hipertensão seja conhecida - mais ativos fisicamente têm menor prevalência de HA -, há recentemente o interesse pelo papel das dimensões da AF. Nesse sentido, evidências acerca das dimensões têm sido mostradas: indivíduos ativos no trabalho, lazer e em práticas esportivas apresentam menor prevalência de HA;^7^ ademais, a insuficiência de AFO e AFL aumenta significantemente o risco de HA.^8^ Entretanto, as atividades físicas ocupacionais, consideradas pesadas, também têm sido associadas à maior prevalência de hipertensão. No entanto, a associação positiva da AFO mais alta com a hipertensão tem sido atribuída, por alguns, ao papel de confundidores não avaliados, dentre eles, as demandas psicológicas.^9-11^ Além disso, tem sido discutido o chamado “paradoxo da AF”, segundo o qual os benefícios da atividade no lazer poderiam ser minimizados pela alta AFO.^12-14^ Portanto, há confluências e lacunas acerca do papel das diferentes dimensões da AF para a ocorrência de HA.

Assim, o objetivo deste estudo foi verificar como se dá a associação das dimensões da AF (AFO, AFL e AFD) isoladamente e de forma cumulativa na ocorrência de HA.

## Método

### Delineamento e população do estudo

Estudo de corte transversal com trabalhadores da Indústria de Calçados (n=446) e trabalhadores da Limpeza Urbana (n=624), totalizando 1.070 participantes. No conjunto dos dois inquéritos, a taxa de resposta foi de 97%. O plano amostral na Indústria de Calçados foi de uma amostra aleatória estratificada proporcional ao sexo e número de trabalhadores de cada uma das duas fábricas no interior da Bahia; na Limpeza Urbana fez-se um censo na empresa municipal em Salvador. A coleta dos dados foi realizada em 2012 e 2010, respectivamente. Como critério de inclusão, todos os trabalhadores entrevistados deveriam estar empregados durante a coleta de dados, que foi conduzida por equipe treinada, composta por profissionais da saúde, segurança e acadêmicos do curso de Fisioterapia. Todos estavam cientes da necessidade de esclarecer dúvidas sobre as perguntas, para obterem respostas as mais fidedignas possíveis. As entrevistas foram realizadas em cada empresa participante, durante o dia de trabalho regular, em local reservado, assegurando a privacidade dos trabalhadores.

### Questionário

O questionário aplicado pelo entrevistador incluiu perguntas sobre: fatores sociodemográficos, características do trabalho e hábitos de vida. Fatores sociodemográficos: sexo, idade, situação conjugal, escolaridade, cor ou raça, e ter ou não filhos. Características do trabalho: demandas físicas, avaliadas através do manuseio de cargas: levantar, empurrar e puxar; aspectos psicossociais do trabalho, medidos por meio do **Job Content Questionnaire** (JCQ), instrumento que mede as dimensões do Modelo Demanda-Controle;^15^ horário de trabalho e tempo de trabalho na empresa estudada. Hábitos de vida: tabagismo atual ou pregresso e frequência de uso de bebida alcoólica. Foram realizadas medidas diretas da PA, peso, altura e circunferência de cintura.

### Variável dependente

A variável dependente é a HA, definida por duas medidas da PA: a primeira, no início da entrevista, com os participantes sentados durante cinco minutos antes da aplicação do questionário e, a segunda, ao final do preenchimento do questionário aplicado pelo entrevistador, com intervalo médio de 20 minutos entre essas tomadas. Foi utilizado um esfigmomanômetro aneroide devidamente calibrado e um estetoscópio. As medidas foram realizadas conforme recomendações da 6ª Diretriz Brasileira de Hipertensão Arterial. Foram considerados casos de hipertensão arterial aqueles com pressão arterial sistólica ≥ 140 e ou pressão arterial diastólica ≥ 90, bem como indivíduos em tratamento regular para a hipertensão. As informações foram dicotomizadas, classificando os indivíduos como aqueles que apresentavam ou não um fenômeno hipertensivo.

### Variável independente principal

A variável independente principal é a AF em três dimensões: ocupacional (AFO), doméstica (AFD) e de lazer (AFL).

A AFO foi obtida por meio do autorrelato do trabalhador sobre suas demandas físicas ocupacionais de manuseio de cargas: levantar, empurrar e puxar, utilizando-se uma escala de duração de 0 a 5, onde 0 representou a ausência de exposição (nunca) e 5, a exposição máxima (o tempo todo). As três variáveis foram somadas e esse valor dividido por três, obtendo-se a média dos escores. Os que atingiram escore ≤ 2 (menos ativos) foram os considerados expostos e os não expostos tiveram escore 3 até 5 (mais ativos).

Em relação à AFL, foi perguntado: “O que você faz quando não está trabalhando na empresa ou em casa?”, com quatro opções de resposta: (I) Conversa com os parentes ou amigos, lê jornal ou revista, vê televisão e vai ao culto (ou missa); (II) caminha, pesca, cuida da horta ou do quintal; (III) corre, faz ginástica, nada, joga bola e anda de bicicleta; (IV) treina para competições. Consideraram-se indivíduos com respostas “I” e “II”, (portanto, com AFL mais baixa), os expostos; e os que responderam positivamente às afirmativas “III” e “IV” foram os considerados ativos no lazer, ou seja, os não-expostos.

Sobre o domínio da AFD, perguntou-se sobre horas semanais dedicadas ao trabalho doméstico. A classificação empregada foi a de expostos e não expostos, ausência de horas dedicadas aos trabalhos domésticos e presença, respectivamente.

Na análise multivariada, utilizou-se a variável principal AF, reunindo as três dimensões em três estratos. Os trabalhadores que tinham alta AF nas três dimensões, ou seja, ativos no lazer, no trabalho e em casa, foram considerados os não expostos, e dois estratos de exposição foram utilizados: o primeiro deles incluiu indivíduos ativos em duas dimensões e, ao segundo estrato, foram incorporados indivíduos inativos ou ativos em apenas uma dimensão.

### Covariáveis

Para a variável idade, adotou-se, como exposto, aqueles trabalhadores com idades acima da mediana. Cor ou raça foi estratificada em “não preta” que incluía branca, parda, amarela e indígena, classificados como não-expostos, e os indivíduos de cor ou raça “preta” foram classificados como expostos. Quanto à escolaridade, os expostos foram os que não tinham o Ensino Médio completo. Para estado civil, foram considerados expostos os que tinham parceiro estável, mesmo que não casados formalmente.

A variável “tipo de jornada” consiste em turno rotativo, grupo exposto, e horário administrativo, os não-expostos. Para os aspectos psicossociais do trabalho, foram utilizadas questões das dimensões demanda psicológica e controle sobre o trabalho, do JCQ. Os indivíduos expostos foram os que possuíam alta demanda e baixo controle sobre o trabalho. Para a variável tempo de trabalho na empresa, os expostos foram os que apresentavam valor acima da mediana.

Considerou-se exposto o indivíduo que fuma ou já foi fumante no passado. Para “frequência do uso de bebida”, os que consumiam álcool mais de uma vez por semana foram os expostos.

O indicador antropométrico utilizado para verificar a associação com HA, na análise multivariada, foi o Índice de Massa Corporal (IMC), calculado a partir das medidas diretas de altura e peso dos indivíduos. Classificou-se o excesso de peso a partir do ponto de corte de ≥ 25 kg/m^2^, e esses foram os expostos, incluindo, portanto, aqueles com sobrepeso e obesidade.^16^

### Análise estatística

Todas as variáveis passaram por uma etapa descritiva e suas frequências foram verificadas. Nas variáveis contínuas, foram feitas medidas de tendência central, de dispersão e posição. Cálculos das razões de prevalência para a HA, segundo as variáveis independentes, foram conduzidas inicialmente na etapa univariada da análise. Na análise multivariada, as covariáveis entraram nos modelos por blocos, tendo como variável independente principal a AF com três estratos: ativos nas três dimensões, ativos em duas, e inativos ou ativos em uma dimensão. O grupo de referência foram os ativos nas três dimensões da AF. A seleção das covariáveis para entrada nos modelos multivariados baseou-se na plausibilidade teórica e biológica e na força de associação bruta da análise univariada. Utilizou-se a Regressão de Cox para estudos transversais.^17^ Testou-se a interação em modelo aditivo das três dimensões da AF para a ocorrência da HA.

A população de estudo, constituída de um *pool* de inquéritos, não foi aleatória. Assim, as análises não utilizaram inferência estatística; ao contrário, o modelo final na análise multivariada é apresentado com todas as variáveis selecionadas, com suas respectivas medidas de associação (Razões de Prevalência) e, portanto, foram todas mantidas no modelo final. Assim, no presente estudo, foram adotados procedimentos compatíveis com a natureza não aleatória da população investigada, de acordo com a ampla literatura sobre o tema.^18,19^ Utilizou-se o programa SPSS 24 (Statistical Package for Social Sciences) para todas as análises.

### Vieses

Com o intuito de minimizar o efeito do trabalhador sadio, trabalhadores selecionados que estavam sob licença médica foram convidados a participar, exceto quando o afastamento estava relacionado à licença maternidade ou decorria de agravo supostamente não relacionado à exposição ocupacional. Nesses casos, o próximo da lista era selecionado em substituição. Vieses de informação foram minimizados com o esclarecimento aos trabalhadores de que os questionários ficariam sob responsabilidade da Universidade Federal da Bahia. Consequentemente, o acesso às informações individuais seria impossibilitado tanto para as empresas como para os gestores.

### Aspectos de ética em pesquisa

Este estudo foi submetido ao Comitê de Ética em Pesquisa da Universidade Federal da Bahia e aprovado, sob parecer n° 1.621.917. Aos participantes foram esclarecidos os objetivos da pesquisa e todos assinaram o Termo de Consentimento Livre e esclarecido.

## Resultados

Ao todo, 1.070 trabalhadores foram incluídos no estudo, sendo que 842 (78,7%) eram homens. A prevalência de HA foi de 24%. Trabalhadores que não concluíram o ensino médio correspondiam a 46% da população, 82% tinham parceiros estáveis e 63% possuíam jornada diária de trabalho com horário de turno. Os que fumam ou já fumaram representaram 26%, e 42% da população consumia bebida alcoólica mais de uma vez por semana. Excesso de peso esteve presente em 43% dos participantes. Nas variáveis da AF, 47% e 61% da população tinham baixa AFO e baixa AFL, respectivamente; 28% não faziam AFD. Idade e IMC apresentaram a maior força de associação com a HA na etapa univariada (RP=2,9 e RP =2,8, respectivamente). Também apresentaram forte associação: sexo (RP=1,95), AFD (RP=1,62) e tipo de jornada (RP=1,60) (Tabela 1).

**Tabela 1:** Fatores associados à hipertensão arterial em trabalhadores da Limpeza Urbana e Indústria de Calçados

Variável	Frequência N	%	Prevalência (%)	RP*
HA sim não	256 812	23,8 76,2		
Sexo mulher homem	228 842	21,3 78,7	13,6 26,6	1 1,95
Idade (anos) ≤ 31 anos > 31 anos	575 491	53,9 46,1	12,7 36,9	1 2,90
Cor ou raça não preta preta	578 489	54,2 45,8	24,0 23,8	1 0,99
Escolaridade ≥2º grau completo < 2º grau completo	576 490	54 46	22,5 25,6	1 1,14
Vive junto não sim	188 880	17,6 82,4	23,5 23,9	1 1,02
Trabalho de alta exigência não exposto exposto	714 326	68,7 31,3	23,4 25,6	1 1,09
Tempo de ocupação ≤3 anos >3 anos	506 562	47,4 52,6	19,4 27,8	1 1,43
Tipo de jornada horário administrativo turno rotativo	392 676	36,7 63,3	17,3 27,6	1 1,59
Fuma ou já fumou não sim	779 270	74,3 25,7	21 31,1	1 1,48
Frequência uso de bebida ≤ 1 vez por semana > 1 vez por semana	616 438	58,4 41,6	18,7 28,8	1 1,44
Excesso de peso não sim	612 455	57,4 42,6	13,5 37,4	1 2,775
AF ocupacional alta baixa	567 501	53,1 46,9	22,0 25,9	1 1,18
AF lazer alta baixa	413 655	38,7 61,3	20,9 25.7	1 1,23
AF doméstica faz não faz	761 300	71,7 28,3	20,3 33	1 1,62

*RP: razão de prevalência; HA: hipertensão arterial; AF: atividade física.

A prevalência de HA por faixa de idade foi: < 35 anos (n=713 indivíduos) 16% de HA, de 35 a 44 anos (n= 262) 37%, de 45 a 54 anos (n=81) 51%, e para > 54 anos (n=7 indivíduos) a prevalência foi de 43% (N= 7). A frequência de HA para cada um dos estratos de AF foi: 16% para indivíduos ativos em três dimensões, 39% para ativos em duas e 45% para inativos ou ativos em apenas uma dimensão (dados não mostrados).

Na tabela 2, está a análise multivariada, com a variável independente principal (AF) em suas combinações. O modelo 1 traz a associação bruta entre AF e HA; no modelo 2, a variável independente principal é ajustada pelas variáveis sociodemográficas e, no terceiro modelo, faz-se ajuste adicional pelas variáveis de estilo de vida, quando se observa um ajuste de 11% na associação principal. A entrada das variáveis ocupacionais no quarto modelo praticamente não alterou a RP da associação principal. Os ativos em duas das três dimensões e os inativos ou ativos em apenas uma dimensão da AF tiveram RP de 1,25 e 1,62, respectivamente. As covariáveis excesso de peso (RP: 2,26), idade > 31 anos (RP: 2,10) e sexo masculino (RP:1,62) mantiveram sua associação com a HA, no modelo final. Não foi encontrada interação no modelo aditivo entre as três dimensões da AF para ocorrência da HA.

**Tabela 2:** – Razões de prevalência entre a AF, combinando as três dimensões, e HAS, em modelos ajustados por variáveis sociodemográficas, de estilo de vida e ocupacionais

Variáveis	mod 1* RP	mod 2* RP	mod 3* RP	MOD 4* RP
AF Ativo em 3 dimensões Ativo em 2 dimensões Inativo ou ativo em 1 dimensão	1,00 1,22 1,76	1,00 1,26 1,80	1,00 1,24 1,60	1,00 1,25 1,62
Sexo feminino masculino		1 1,94	1 1,66	1 1,62
Idade ≤ 31 anos > 31 anos		1 2,63	1 2,22	1 2,10
Excesso de peso não sim			1 2,24	1 2,26
Fuma ou já fumou não sim			1 1,11	1 1,11
Frequência de bebida ≤ 1 vez por semana > 1 vez por semana			1 1.18	1 1,18
Tipo de jornada horário administrativo turno				1 1,05
Tempo de trabalho ≤3 anos >3 anos				1 1,17

*MOD 1: associação bruta; MOD 2: ajustado por sexo e idade; MOD 3: ajustado por excesso de peso, se fuma ou já fumou e frequência de bebida; MOD 4: ajustado por tipo de jornada e tempo de trabalho; RP: razão de prevalência. AF: atividade física

## Discussão

O acúmulo de AF nas dimensões da AFO, AFD e AFL pode resultar em melhorias da HA. Ser ativo em uma ou nenhuma das três dimensões da AF demonstrou maior associação positiva com o desfecho: 62% mais HA comparados àqueles ativos nas três dimensões. Esses achados contribuem com a literatura sobre o papel da AF insuficiente como fator de risco modificável para HA, e coloca em questão o papel isolado da AFL como estratégia para o controle desse agravo.

No que diz respeito à cor ou raça, a forte miscigenação da população na Bahia pode estabelecer limites para investigar sua associação com a HA. Conforme descrito por outros autores,^20^ a provável homogeneização dos grupos, pela forte miscigenação, deve ter um papel no resultado que revelou prevalências de HA praticamente iguais entre os grupos “pretos” e “não pretos”, nessa população.^21,22^ Em consonância com essa hipótese explicativa, um estudo multicêntrico sobre HA que, além de servidores da Universidade Federal da Bahia, incluiu servidores de mais cinco instituições do Brasil, de diferentes estados da Federação,^23^ encontrou forte associação dessa variável com a HA, cujas prevalências são maiores em pretos, seguidos dos pardos, sendo a menor prevalência observada entre os brancos.

A associação da HA com idade e sexo foi bem consistente com a literatura.^11,23-26^ Essa população é composta por 79% de homens jovens, com 32 anos de idade média. É visto que, nessa fase da vida, os homens apresentam mais HA quando comparados às mulheres.^1^ Estudos que revelam maior prevalência entre mulheres são baseados em autorrelato ou populações em faixas etárias mais elevadas, nas quais o sexo feminino supera o masculino com maior prevalência.^27^ Quanto aos estudos baseados em autorrelato,^1^ a predominância de HA entre mulheres pode ser decorrente da maior busca de serviço por essas e, consequentemente, maior conhecimento do estado hipertensivo.

No Brasil, em 2016, a prevalência de HA aumentou com a idade, sendo de 19,1% naqueles indivíduos entre 35 e 44 anos.^1^ Comparando com os resultados do presente estudo, viu-se que, dentre indivíduos na faixa etária de 35 a 44 anos, a prevalência de HA é de 37%. Portanto, entre os aqui estudados, a HA foi mais alta do que a média nacional. Em comparação com Chor et al.,^23^ que estudaram servidores de universidades, os estratos por idade deste estudo com operários apresentaram prevalências mais altas.

Operários com excesso de peso tiveram 2,3 vezes maior prevalência de HA quando comparados com aqueles sem excesso de peso. Esse resultado é consistente com outros estudos, demonstrando associação da HA com maior densidade corporal.^28,29^ As demais variáveis que se associaram na etapa univariada reduziram muito suas associações com HA na análise multivariada.

A variável independente principal associou-se consistentemente com a HA na análise multivariada, constatando-se efeito cumulativo da baixa AF nas três dimensões sobre a prevalência da HA.

Explorou-se a associação independente entre atividade física ocupacional (AFO) e HA por meio de análises que contemplaram AFO tanto com escala de duração quanto de intensidade do manuseio de carga. Os trabalhadores mais ativos nessa dimensão foram mais protegidos para HA, mesmo com ajustes para idade, sexo e excesso de peso. A motivação para essas análises com a AFO baseou-se na recente discussão da literatura acerca do “efeito paradoxal” da AFO sobre a pressão arterial.^30^ Segundo pesquisadores, indivíduos submetidos à alta AFO apresentariam maior risco para níveis pressóricos elevados.^14,31^ É possível que ocupações que se caracterizam por recrutamento de grandes grupos musculares, a exemplo da coleta de lixo, embora possam determinar fadiga física, não parecem representar associação com HA, como proposto no efeito paradoxal. No presente estudo, o trabalho ativo foi sempre protetor para esse agravo, particularmente, quando associado à AFD e AFL.

Embora discuta-se a AFL como um dos principais tratamentos não farmacológicos contra a HA,^32-34^ de acordo com o presente estudo, a AFL isolada pode não suprir a insuficiência de AF para o melhor controle da HA: ser ativo em apenas uma dimensão, e inativo nas duas demais dimensões, apresentou associação positiva com a HA.

Poucas pesquisas exploram a AFD de forma isolada e resultados com essa dimensão parecem não mostrar associação com HA. Porém, quando combinada com a prática de AF nas outras dimensões, a AFD pode produzir efeitos benéficos no controle da HA.

Embora no presente estudo tenha sido observado efeito cumulativo da baixa AF nas diferentes dimensões para a ocorrência da HA, não foi encontrada interação entre essas dimensões (nem no modelo aditivo). Fato semelhante foi observado em outro estudo, que também revelou efeito cumulativo das diferentes dimensões, mas não interação entre essas.^7^ Análise de interação para explorar o comportamento sedentário e ou tempo de inatividade em frente à TV na determinação de doenças crônicas em adultos parece uma abordagem promissora.^35^

Além do acúmulo de AF nas diversas dimensões ser um fator de proteção, o acúmulo durante os vários estágios da vida pode melhorar ainda mais os indicadores de saúde.^36^

Neste estudo, a AF foi obtida pelo autorrelato, e não medida de forma direta, através de monitores cardíacos, acelerômetros, pedômetros ou frequencímetros. Apesar da tendência em se considerar que medidas diretas sejam mais válidas do que o autorrelato, essas têm sido questionadas enquanto padrão-ouro na Epidemiologia Ocupacional. Devido ao seu custo e exigências de calibração de instrumentos, medidas diretas são obtidas, em geral, durante pequenas amostras de tempo e, no caso da AFO, em uma pequena amostra da jornada de trabalho. Isto é particularmente relevante, limitando a validade das medidas diretas, em estudos com populações cujas atividades ocupacionais apresentem grande variabilidade durante a jornada de trabalho e, nesse caso, a amostra de tempo da jornada no qual se fazem as medidas diretas não é representativa do tempo total de trabalho.^37^

No delineamento do estudo de corte transversal, o principal viés é o de prevalência e, portanto, não se pode descartar a causalidade reversa, ou seja, não se pode afirmar rigorosamente se a maior prevalência de HA dos menos ativos é decorrente do estilo de vida sedentário ou se eles já eram hipertensos antes de passarem a ter uma vida mais sedentária. Porém, como a HA é um agravo crônico, cuja terapêutica assegura seu controle, mas não a cura, é possível que esse tipo de viés esteja minimizado, sendo o corte transversal uma opção adequada de delineamento de estudo. A população do estudo é composta predominantemente por jovens trabalhadores, mas os resultados obtidos e descritos de prevalência de hipertensão para as diferentes faixas etárias permitem comparação com outras populações, desde que observada cada faixa de idade.

## Conclusão

Neste estudo, evidenciou-se que o menor volume de movimento nas diversas dimensões da AF foi um fator associado à HA nessa população. Visto que outros estudos demonstram linha semelhante de evidências, os resultados do presente estudo podem ser considerados válidos. A partir destes, considerou-se que a investigação da AF, fator modificável na ocorrência da HA, não pode prescindir da abordagem das suas diferentes dimensões, sob pena de implicar em erro metodológico ao classificar sujeitos ativos no trabalho ou no ambiente doméstico como inativos, ao se basear apenas na dimensão do lazer. Devem ser planejadas intervenções no ambiente ocupacional, objetivando ouvir dos trabalhadores o significado da HA para eles próprios, dando acesso aos resultados de pesquisas, a fim de estimular mudanças de hábitos e a ampliação do protagonismo dos trabalhadores na definição do lazer ativo, além de dar visibilidade à importância da AFO. Novas investigações devem aprimorar o estudo da AF, a fim de trazer novas evidências sobre seu papel na HA.
