# Supplementary material for: Hipertensão Arterial em Trabalhadores: O Efeito Cumulativo das Dimensões da Atividade Física sobre esse Agravo
Source: Arq Bras Cardiol. 2020 May 22;114(5):755–61. [Article in Portuguese] doi: 10.36660/abc.20190065 (PMC8386995; doi:10.36660/abc.20190065)
Supplement: Translation

# Translation: Hypertension in Workers: The Role of Physical Activity and its Different Dimensions

**Authors:** Uelito Everaldo Souza Ribeiro, Rita de Cassia Pereira Fernandes

**Affiliations:** 1 Brazil Universidade Federal da Bahia - Programa de Pós-Graduação em Saúde, Ambiente e Trabalho, Salvador, BA - Brazil; 2 Brazil Universidade Federal da Bahia - Faculdade de Medicina da Bahia, Salvador, BA – Brazil

**Keywords:** Hypertension, Workers, Urban Cleaning Service, Motor Activity, Anthropometry, Physical Exerton, Work, Socioeconomic Factors, Life Style

## Abstract

**Background:**

Physical activity, each type in its own manner, whether occupational, domestic or leisure, can play a significant role regarding high blood pressure (HBP). However, practicing physical activity only at leisure time, or in specific situations, can be insufficient to achieve the effective control of HBP.

**Objective:**

To analyze the isolated and cumulative effect of different types of physical activity and the prevalence of HBP among workers.

**Methods:**

A cross-sectional study with 1,070 Urban Cleaning and Footwear Industry workers in Bahia, who answered a survey, conducted by an interviewer on sociodemographic, occupational, lifestyle and hypertensive morbidity aspects. Weight, height, waist circumference and blood pressure were measured. Case of HBP: Systolic blood pressure ≥140 or diastolic blood pressure ≥90, or regular treatment for HBP. The occupational, domestic and leisure aspects of Physical Activity were studied. A multivariate analysis with Cox Regression was performed for cross-sectional studies.

**Results:**

The prevalence of HBP was 24%, being 37% among workers aged between 35-44 years, and 51% among workers aged between 45-54 years. The multivariate model showed that workers who were active in one form of physical activity only or no forms had 62% higher BP levels and that these levels were 25% higher among workers who were active in two out of three physical activity forms. Being a male, being older (> 31 years old) and being overweight were characteristics associated with HBP, with prevalence ratios of 1.62, 2.10 and 2.26, respectively.

**Conclusions:**

There was a cumulative effect of the form of physical activity on the occurrence of HBP. Classifying active subjects at work or at home as inactive persons by relying only on the leisure form can lead to methodological errors. (Arq Bras Cardiol. 2020; 114(5):755-761)

## Introduction

High blood pressure (HBP) is a chronic disease defined by blood pressure levels greater than or equal to 140/90 mmHg and represents one of the risk factors for cardiovascular diseases. On the other hand, it is known that insufficient or lack of physical activity (PA) is a modifiable risk factor for this condition.^1,2^

Every movement produced by skeletal muscles that generates energy greater than the resting state is considered PA. An active person is an individual who practices at least 30 minutes of moderate-intensity PA five days a week. This does not include leisure only; on the contrary, the literature emphasizes the importance of approaching PA in different forms: leisure (PA-L), occupational (PA-O), domestic (PA-D) and displacement.^3,4^

Brazil, a signatory to the World Health Organization’s Plan for the Prevention of Noncommunicable Chronic Diseases, committed to a 25% relative reduction in the prevalence of HBP and a 10% reduction in insufficient PA by the year 2025. This challenge highlights the relevance of HBP as a health problem.^5,6^

Although a negative association between PA and HBP has been observed - more physically active people have lower HBP prevalence - there is a recent interest in the role of PA forms. In this sense, evidence about the forms has shown that individuals who are active at their work, leisure time and also practice sports have lower prevalence of HBP;^7^ in addition, the lack of PA-O and PA-L significantly increases the risk of HBP.^8^ However, occupational physical activities, considered strenuous, have also been associated with higher prevalence of hypertension, but this positive association of higher PA-O with hypertension has been attributed by some researchers to the role of unvalued confounders, such as psychological demands.^9 -11^ In addition, the so-called “PA paradox” has been discussed, according to which the benefits of leisure activity could be minimized by high levels of PA-O.^12-14^ Therefore, there are confluences and gaps regarding the role of the different PA forms on the occurrence of HBP.

Thus, the objective of this study was to verify how forms of PA (PA-O, PA-L and PA-D) are associated with the occurrence of HBP, both alone and cumulatively.

## Method

### Study design and population

A cross-sectional study with Footwear Industry workers (n=446) and Urban Cleaning workers (n=624), totaling 1,070 subjects. In both survey clusters, the response rate was 97%. The sampling plan in the Footwear Industry was a stratified random sample proportional to the gender and number of workers from each of the two factories in the countryside of Bahia; in Urban Cleaning, a census was carried out at the municipal company in Salvador. Data collection was carried out in 2012 and 2010. As an inclusion criterion, all workers interviewed should be employed during data collection, which was conducted by a trained team of health and safety professionals and students of the physical therapy program. Everyone was aware of the need to clarify any aspects about the questions in order to obtain the most reliable answers possible. The interviews were carried out in each participating company, during regular working days, in a reserved place, ensuring the workers’ privacy.

### Survey

The survey carried out by the interviewer included questions about sociodemographic factors, job characteristics and life habits. The sociodemographic factors asked were gender, age, marital status, schooling, color or race, and if the interviewed worker had any children. Job characteristics: physical demands, evaluated through the handling of loads (lifting, pushing and pulling); psychosocial aspects of work, measured using the Job Content Questionnaire (JCQ), an instrument that measures the forms of the Demand-Control Model;^15^ working hours and time of service in the company studied. Life habits: current or previous smoker and frequency of alcohol use. Direct measurements of Blood Pressure (BP), weight, height and waist circumference were performed.

### Dependent variable

The dependent variable is HBP, defined by two measures of BP: the first at the beginning of the interview, with subjects sitting for five minutes before the start of the survey, and the second upon completion of the survey, as carried out by the interviewer, with a mean interval of 20 minutes between these measurements. A duly calibrated aneroid sphygmomanometer and stethoscope were used. The measurements were performed according to the recommendations of the Sixth Brazilian Guidelines on High Blood Pressure. Cases of hypertension were considered as those with systolic blood pressure ≥ 140 and/or diastolic blood pressure ≥ 90, and subjects undergoing regular treatment for hypertension. The information was divided into two groups, classifying subjects as those who did and those who did not show a hypertensive condition.

### Main independent variable

The main independent variable is PA in three forms: occupational (PA-O), domestic (PA-D) and leisure (PA-L).

The level of PA-O was obtained through the worker’s self-report on his physical demands of load handling (lifting, pushing and pulling), using a 0-5 scale, where 0 meant no exposure (never) and 5 meant maximum exposure (all the time). The three variables were summed, with the resulting value divided by three, to calculate the mean of the scores. Those workers who reached score ≤ 2 (less active) were considered exposed, whereas the non-exposed ones had scores ranging from 3 to 5 (more active).

For the PA-L, the following question was asked, “What do you do when you’re not working at home or at work?”, with four answer options: (I) Talk to family or friends, read the newspaper or a magazine, watch TV, go to church; (II) Hike, fish, maintain the garden or the yard; (III) Run, exercise, swim, play a ball game, ride a bicycle; (IV) Train for a competition. Individuals with “I” and “II” responses were considered, therefore, to have lower PA-L levels, i.e., the exposed ones; and those who responded positively to “III” and “IV” were considered active in leisure, i.e., the non-exposed ones.

In the PA-D domain, workers were questioned about weekly hours devoted to domestic work. They were classified as exposed and non-exposed, absence and presence of hours dedicated to housework, respectively.

In the multivariate analysis, the main variable PA was used, subdividing the three forms into three strata. Workers who had high PA levels in the three forms, i.e., active at leisure, at work and at home, were considered non-exposed, and two strata of exposure were used: the first one included active individuals in two forms; and the second, individuals who were inactive or active in only one form.

### Covariables

For the age variable, those workers who were older than the median age were considered to be exposed. Color or race was stratified into “non-black”, which included White, Brown, Asian and Indigenous, classified as non-exposed, whereas Black individuals were classified as exposed. As for schooling, the exposed ones were those who had not completed High School. For marital status, those who had a stable partner were exposed, even if they were not formally married.

The variable “type of workday” consists of rotating shifts (exposed groups) and business hours (non-exposed groups). For the psychosocial aspects of work, psychological demand and job control questions, from the JCQ, were used. Exposed individuals were those who had high demand and low control over their work. For the time of service variable, those exposed were the ones with values above the median.

An individual who smokes or used to smoke was considered exposed. For “alcohol use frequency”, those who consumed alcohol more than once a week were classified as exposed.

The anthropometric indicator used to verify the association with HBP in the multivariate analysis was Body Mass Index (BMI), calculated from subjects’ direct height and weight measurements. Excess weight was considered present above the cut-off point of ≥ 25 kg/m^2^, and those were the exposed ones, including, therefore, those who were overweight and obese.^16^

### Statistical analysis

All variables went through a descriptive analysis and their frequencies were verified. For continuous variables, measures of central tendency, dispersion and position were provided. The HBP prevalence ratio, according to the independent variables, were was initially calculated in the univariate analysis. In the multivariate analysis, the covariables entered the block models, and PA was considered as a main independent variable with three strata: active in the three forms, active in two forms, and inactive or active in one form. The reference group consisted of active individuals in the three PA forms. The selection of covariables for entry into the multivariate models was based on the theoretical and biological plausibility, and on the gross association strength of the univariate analysis. The Cox regression model was used for cross-sectional studies.^17^ Interaction was tested using an additive model of the three PA forms for the occurrence of HBP.

The study population, consisting of a pool of surveys, is not random. Thus, the analyses did not use statistical inference; on the contrary, the final model in the multivariate analysis is presented with all the selected variables, with their respective association measures (Prevalence Ratios). For this reason, they are all kept in the final model. Thus, in this study, procedures compatible with the non-random nature of the population investigated were used, according to an extensive literature on the subject.^18,19^ All analyses were done using the Statistical Package for the Social Sciences (SPSS) software, version 24.0.

### Biases

In order to minimize the effect of healthy workers, selected workers who were on medical leave were invited to participate, except when the leave was maternity-related or was due to an injury allegedly unrelated to occupational exposure. In this case, the next one on the list would be selected instead. Information biases were minimized with clarifications being provided to workers. The surveys would be under the responsibility of the Federal University of Bahia, so that it would be impossible for both companies and managers to access individual information.

### Aspects of research ethics

This study was submitted to the Research Ethics Committee of the Federal University of Bahia and approved under opinion No. 1,621,917. Subjects were notified about the objectives of the research and all participants signed an Informed Consent Form.

## Results

A total of 1,070 workers were studied, of which 842 (78.7%) were men. The prevalence of HBP was 24%. Workers who did not complete High School made up 46% of the population, 82% had stable partners, 63% had daily working hours with shift schedules. Those who smoke or have smoked accounted for 26%, and 42% of the population consumed alcohol more than once a week. Overweight was present in 43% of the subjects. In PA variables, 47% and 61% of the population had low PA-O and PA-L levels, respectively; 28% were not involved in PA-D. Age and BMI had the greatest factor associated with HBP in the univariate analysis (PR = 2.9 and PR = 2.8, respectively). The following also had strong association: gender (PR = 1.95), PA-D (PR = 1.62) and type of workday (PR = 1.60) (Table 1).

**Table 1:** Factors associated with high blood pressure in Urban Cleaning and Footwear Industry Workers

Variable	Frequency N	%	Prevalence (%)	PR*
HBP yes no	256 812	23.8 76.2		
Gender male famale	228 842	21.3 78.7	13.6 26.6	1 1.95
Age ≤ 31 year > 31 year	575 491	53.9 46.1	12.7 36.9	1 2.90
Color or race Non-black Black	578 489	54.2 45.8	24.0 23.8	1 0.99
Schooling ≥ high school < high school	576 490	54 46	22.5 25.6	1 1.14
hade a stable partner no yes	188 880	17.6 82.4	23.5 23.9	1 1.02
Higt-demand work non-exposed exposed	714 326	68.7 31.3	23.4 25.6	1 1.09
time of service ≤3 year >3 year	506 562	47.4 52.6	19.4 27.8	1 1.43
Type of workday business hours rotating shift	392 676	36.7 63.3	17.3 27.6	1 1.59
Smokes or used to smoke no yes	779 270	74.3 25.7	21 31.1	1 1.48
frequency of alcohol use ≤ 1 once a week > 1 once a week	616 438	58.4 41.6	18.7 28.8	1 1.44
Excess weight no yes	612 455	57.4 42.6	13.5 37.4	1 2.775
PA occupational high low	567 501	53.1 46.9	22.0 25.9	1 1.18
PA leisure high low	413 655	38.7 61.3	20.9 25.7	1 1.23
PA domestic yes no	761 300	71.7 28.3	20.3 33	1 1.62

* PR: prevalence ratio; HBP: high blood pressure; PA: physical activity.

The prevalence of HBP by age range was: <35 years (n = 713 individuals) 16% of HBP, 35-44 years (n = 262) 37%, 45-54 years (n = 81) 51%, and for > 54 years (n = 7) individuals, the prevalence was 43%. The frequency of HBP for each one of the PA strata was: 16% for individuals who were active in the three forms, 39% for those who were active in two forms, and 45% for individuals who were inactive or active in only one form (data not shown).

Table 2 shows the multivariate analysis, with the main independent variable, PA, in its combinations. Model 1 shows the gross association between PA and HBP. In model 2, the main independent variable is adjusted by sociodemographic variables. And in model 3, an additional adjustment is made for lifestyle variables, when an adjustment of 11% in the main association is observed. The entry of occupational variables in model 4 practically did not change the PR of the main association. Active individuals in two of the three forms, and inactive or active individuals in only one form of PA had a PR of 1.25 and 1.62, respectively. Overweight (PR: 2.26), age > 31 years (PR: 2.10) and male gender (PR: 1.62) covariables maintained their association with HBP in the final model. No interaction was found in the additive model between the three forms of PA for the occurrence of HBP.

**Table 2:** – Prevalence ratios between PA, combining the three forms, and systemic hypertension, in models adjusted for sociodemographic, lifestyle and occupational variables

Variable	mod 1* PR	mod 2* PR	mod 3* PR	MOD 4* PR
PA active in 3 dimensions active in 2 dimensions inactive or active in 1 dimensions	1.00 1.22 1.76	1.00 1.26 1.80	1.00 1.24 1.60	1.00 1.25 1.62
Gender male famale		1 1.94	1 1.66	1 1.62
Age ≤ 31 year > 31 year		1 2.63	1 2.22	1 2.10
Excessive weight no yes			1 2.24	1 2.26
Smokes or used to smoke no yes			1 1.11	1 1.11
frequency of alcohol use ≤ 1 once a week > 1 once a week			1 1.18	1 1.18
Type of workday business hours rotating shift				1 1.05
time of service ≤3 year >3 year				1 1.17

* MOD 1: gross association; MOD 2: adjusted for gender and age; MOD 3: adjusted for overweight, currently smoking or smoked in the past and frequency of alcohol use; MOD 4: adjusted for type of workday and working hours; PR: prevalence ratio; PA: physical activity.

## Discussion

PA accumulation in PA-O, PA-D and PA-L forms can lead to HBP improvements. Being active in one or none of the three forms of PA demonstrated a greater positive association with the outcome: 62% more HBP compared to those who are active in the three forms. These findings contribute to the literature on the role of insufficient PA as a modifiable risk factor for HBP and calls into question the isolated role of PA-L as a strategy to control this condition.

Regarding color or race, the strong miscegenation of the population in Bahia can represent a limitation to investigate its association with HBP. As described by other authors,^20^ the probable homogenization of ethnical groups, due to strong miscegenation, should play a role in the result, which revealed an almost equal prevalence of HBP among the “black” and “non-black” groups in this population.^21,22^ According to this explanatory hypothesis, a multicenter HBP study which, in addition to including servers from the Federal University of Bahia also included servers from five other institutions in different states in Brazil,^23^ found a strong association of this variable with HBP, whose prevalence was higher among Black people, followed by Brown people, with the lowest prevalence among Caucasians.

The association of HBP with age and gender was very consistent with the literature.^11,23-26^ The studied population was composed of 79% young men, with a mean age of 32 years. Men in this stage of life are seen to have higher prevalence of HBP than women.^1^ Studies that show a higher prevalence among women are based on self-report or populations in higher age groups, in which the female gender shows higher values than the male gender with greater prevalence.^27^ As for studies based on self-reporting,^1^ the predominance of HBP among women may be due to the fact that women are more likely than men to seek out health care and, consequently, greater knowledge of their hypertensive condition is available.

In Brazil, in 2016, the prevalence of HBP was increasing with age, and among those aged between 35 and 44 years, the prevalence was 19.1%.^1^ In a comparison with the results of this study, a prevalence of 37% was found in the age group of 35 to 44 years; therefore, among those studied here, HBP was higher than the national average. In comparison with Chor et al.,^23^ who studied university servers, the age strata of this study with workers had higher prevalence rates.

Overweight workers had 2.3 times the prevalence of HBP compared to those who were not overweight. This result is consistent with other studies, demonstrating the association of HBP with greater body density.^28,29^ The other variables that were associated in the univariate stage had their associations with HBP greatly reduced in the multivariate analysis.

The main independent variable was consistently associated with HBP in the multivariate analysis, showing a cumulative effect of low PA in the three forms regarding the prevalence of HBP.

The independent association between occupational physical activity (PA-O) and HBP was explored through analyses that included PA-O, both with a duration scale and an intensity scale of load handling. The most active workers in this form were more effectively protected against HBP, even with age, gender and overweight adjustments. Motivation for these analyses with PA-O levels was based on the recent literature discussion about the “paradoxical effect” of PA-O on blood pressure.^30^ According to researchers, individuals subjected to high PA-O levels would have a higher risk of HBP.^14,31^ It is possible that occupations that demand the use of large muscle groups, such as garbage collection, although they may exhibit physical fatigue, do not seem to show association with HA, as proposed in the paradoxical effect. In this study, active work was always protective against this condition, particularly when associated with PA-D and PA-L.

Although PA-L is deemed to be one of the main non-pharmacological treatments for HBP,^32-34^ according to the present study, PA-L alone may not compensate for insufficient PA for the purposes of effective HBP control. Being active in only one form and inactive in the other two forms showed a positive association with HBP.

Few studies explore PA-D alone. The data on this form of PA do not seem to show an association with HBP. Nevertheless, when combined with the practice of PA in other forms, PA-D can produce beneficial effects on HBP control.

Although the present study showed the cumulative effect of low PA in its different forms as a risk factor for HBP, no interaction was found between these forms (not even in the additive model). A similar fact was observed in another study, which also revealed a cumulative effect of different forms of PA, but no interaction among them.^7^ Interaction analysis to explore sedentary behavior and/or time spent in front of the TV in determining chronic diseases in adults seems to be a promising approach.^35^

In addition to PA accumulation in the different forms being a protective factor, accumulation during the various stages of life can further improve health indicators.^36^

In this study, PA levels were obtained from self-report and not measured directly through cardiac monitors, accelerometers, pedometers and frequency meters. Despite the tendency to consider direct measures more valid than self-reporting, the first have been questioned as the gold standard in Occupational Epidemiology. Due to their cost and instrument calibration requirements, direct measurements are usually obtained from short time sampling and, in the case of PA-O, during a small sample of the workday schedule. This is particularly relevant in studies with populations whose occupational activities involve high variability over the course of a workday, since this factor may limit the validity of direct measurements. In other words, direct measurements that sample a small proportion of the workday may not be representative of the whole working day.^37^

In the cross-sectional study design, the main bias is the prevalence. Therefore, reverse causality cannot be ruled out, i.e., it is not possible to determine whether the higher prevalence of HBP among less active individuals is due to a sedentary life or whether they were already hypertensive before moving on to a more sedentary life. However, since HBP is a chronic disease, treatment can ensure its control, but not its cure. Thus, this type of bias can be minimized, and cross-section may be an appropriate option for study design. The study population is predominantly composed of young workers, but the results obtained and described for the prevalence of HBP among the different age groups allow for comparisons with other populations, provided that each age group is observed.

## Conclusion

This study showed that lower activity levels in the different forms of PA was a risk factor associated with HBP in this population. Since other studies demonstrate a similar line of evidence, the results of this study can be considered valid. From these studies, it was considered that the investigation of PA, a modifiable factor in the occurrence of HBP, cannot waive its different forms, otherwise it would imply a methodological error when classifying subjects who are active at work or at home as inactive individuals, based on the form of leisure. Interventions should be planned in the occupational environment, so workers have the opportunity to express what HBP means for themselves, and get access to study results in order to stimulate changes in habits and to increase the role of workers in electing active leisure activities and highlighting the importance of PA-O. New research should enhance the study of PA in order to bring new evidence on its role in HBP.
